# Nematode genome announcement: The draft genome sequence of entomopathogenic nematode *Heterorhabditis indica*

**DOI:** 10.21307/jofnem-2021-101

**Published:** 2022-01-21

**Authors:** Chaitra G. Bhat, Vishal S. Somvanshi, Roli Budhwar, Jeffrey Godwin, Uma Rao

**Affiliations:** 1 Division of Nematology, ICAR-Indian Agricultural Research Institute, New Delhi 110012, Delhi, India; 2 Bionivid Technology Private Limited, 209, 4th Cross Rd, B Channasandra, Kasturi Nagar, Bengaluru 560043, Karnataka, India

**Keywords:** Draft genome sequence, Entomopathogenic nematode, *Heterorhabditis indica*, Illumina

## Abstract

*Heterorhabditis indica* is one of the most widely used entomopathogenic nematodes for the biological control of agricultural insect pests worldwide. The draft genome of *H. indica* was sequenced using three genomic libraries of 300 bp, 600 bp and 5 kb sizes by Illumina HiSeq platform. The size of the draft genome assembly was 91.26 Mb, comprising 3,538 scaffolds. Genome completeness analysis by BUSCO (Benchmarking Universal Single-Copy Orthologs) showed 84% complete, and 6.5% fragmented BUSCOs. Further, 10,494 protein-coding genes were predicted. The *H. indica* draft genome will enable comparative and functional genomic studies in *Heterorhabditis* nematodes.

Entomopathogenic nematodes (EPNs) of the genera *Heterorhabditis* and *Steinernema* are used worldwide for the biological control of agricultural insect pests (Lacey and Georgis, 2012; Bhat et al., 2020). These EPNs are also an excellent and genetically tractable model to study mutualism and parasitism (Campos-Herrera et al., 2012). Twenty-one *Heterorhabditis* and one hundred *Steinernema* species have been described from various parts of the world (Bhat et al., 2020). However, their full potential as bio-control agents and as a model system remains under-exploited. Omics information such as genome and transcriptome data and genome interrogation and editing techniques such as RNAi and CRISPR-Cas9 are potent methods to explore EPN biology and lay the groundwork for improving their bio-control traits (Lu et al., 2016). Genomic information about EPNs is scant in the public domain. Presently, whole-genome information is available for only one heterorhabditid and seven steinernematid nematodes (Lu et al., 2016; Baniya and DiGennaro, 2021). Here, we present the first draft genome sequence assembly for *Heterorhabditis indica*, which is widely present in the warmer and tropical climatic regions and is one of the most commercialized EPNs (Lacey and Georgis, 2012).

The *H. indica* strain IARI-EPN-Hms1 (Ganguly et al., 2010) was inbred for 20 generations (designated *H. indica* Hms1-i20) to obtain genetically homogenous nematodes for genome sequencing. Inbreeding involved self-fertilizing the hermaphrodites for 20 generations by placing a single L4 nematode onto the lawn of *Photorhabdus akhurstii* on the nutrient-agar medium supplemented with cholesterol. Inbred nematodes were lysed using Qiagen buffer G2 (Catalogue no. 19060, Qiagen, USA), and high molecular weight DNA was extracted using the phenol-chloroform method. DNA concentration and quality were estimated using Qubit 4.0 Fluorometer (ThermoFisher Scientific, USA) and agarose gel electrophoresis (0.6% agarose gel, 120 min run time at 100 V). DNA was fragmented by sonication using Bioruptor (Diagenode, Seraing (Ougrée), Belgium). The size distribution was checked by running an aliquot of the fragmented DNA sample on an Agilent high sensitivity bioanalyzer (Agilent Technologies, Santa Clara, CA, USA). Illumina TruSeq DNA sample preparation guide (Illumina, San Diego, CA, USA) was followed to prepare two high-quality libraries of insert sizes 300 bp and 600 bp. Illumina Nextera Mate Pair Library Prep Kit was used to prepare a mate-pair library of insert size 5 kb. Paired-end reads of length 2*151, and 2*131 (Mate-pair) were generated on Illumina HiSeq 4000 platform.

A total of 22.74 Gb sequence data comprising of ~160 million reads was generated. The reads provided 750X coverage of the *H. indica* genome. Quality filtering of raw reads by fastp version 0.22 (Chen et al., 2018) identified 155 million High Quality (HQ) reads. The raw and filtered read statistics for *H. indica* are presented in Table 1. The HQ reads were used to generate primary assembly using SOAPDenovo assembler Release 1.0 (Li et al., 2010). The contaminating mitochondrial and bacterial sequences (of *Photorhabdus* symbiont) were removed from the assembly using BlobTools v1.0.1 (Laetsch and Blaxter, 2017) and the NCBI server. Primary assembly contigs were further scaffolded using SSPACE ‘Standard’ version (Boetzer et al., 2011) to generate the final draft assembly. The final *H. indica* genome assembly was of 91.26 Mb size having 3,538 scaffolds with an N50 of 587 kb and an average scaffold length of 25.79 kb. The GC content of the assembled genome was 35.31%, and there were 7.33% N’s in the assembly. Assembly statistics for the *H. indica* Hms1-i20 are provided in Table 2. Genome completeness assessment was done by Benchmarking Universal Single-Copy Orthologs (BUSCO) v5.2.2 (Seppey et al., 2019) against the 3,131 BUSCOs in the nematoda_odb10 database. The *H. indica* Hms1-i20 genome showed the presence of 2630 (84%) complete BUSCOs (complete and single-copy- 2617 (83.6%); complete and duplicated- 13 (0.4%)), whereas 205 (6.5%) BUSCOs were fragmented, and 296 (9.5%) were missing. Genome completeness using Core Eukaryotic Genes Mapping Approach (CEGMA) (Parra et al., 2007) against 248 ultra-conserved core eukaryotic genes showed 97.17% completeness. A total of 10,494 protein-coding genes were predicted in the *H. indica* Hms1-i20 by SNAP (Korf, 2004), Augustus (Stanke and Morgenstern, 2005), GeneMark (Besemer and Borodovsky, 2005), and Maker (Cantarel et al., 2008). Functional annotation by NCBI Blastx+/RefSeq/SwissProt/UniProt databases resulted in the annotation of 9,596 genes. Identification of orthologous genes present in *H. indica* Hms1-i20 genome compared to four other nematode genomes by Orthofinder (Emms and Kelly, 2019) revealed that *H. indica* shared 2,848 groups with *H. bacteriophora*, 4,526 with *C. elegans*, 4,059 with *S. carpocapsae* and 3,396 with *Oscheius tipulae*.

**Table 1.** Read statistics for *H. indica* Hms1-i20 genome.

	Raw reads			Filtered high quality (HQ) reads			
Genomic library (Insert size)	Total reads (million)	Total bases (Giga bases)	GC content (%)	Total HQ reads (Million)	Total HQ bases (Giga bases)	GC content (%)	% HQ reads
300 bp	48.37	7.30	40.92	47.15	7.02	40.62	97.47
600 bp	39.71	5.99	41.12	38.08	5.67	40.89	95.89
5 Kb (mate pair library)	72.27	9.44	39.65	69.75	8.77	38.83	96.51

**Table 2.** Genome assembly statistics of *H. indica* Hms1-i20 genome.

Parameter	Statistics
Total sequences	3,538
Total bases	91,263,125
Min sequence length (bp)	500
Max sequence length (bp)	2,409,384
Average sequence length (bp)	25,795.12
Median sequence length (bp)	3410.50
N50 length (bp)	587,367
As	28.71%
Ts	28.64%
Gs	17.67%
Cs	17.64%
(A + T)s	57.35%
(G + C)s	35.31%
Ns	7.33%

This genomic resource will facilitate functional and comparative genomic studies and genetic exploration in *Heterorhabditis* nematodes.

**Accession number(s):** The raw sequence data has been deposited in GenBank under BioProject No. PRJNA720543, BioSample No. SAMN18671197 and SRA IDs SRR14181568, SRR14181569 and SRR14181570. This Whole Genome Shotgun project has been deposited at DDBJ/ENA/GenBank under the accession JAJAVP000000000. The version described in this paper is version JAJAVP010000000.
